# The Cyclic Nitroxide TEMPOL Ameliorates Oxidative Stress but Not Inflammation in a Cell Model of Parkinson’s Disease

**DOI:** 10.3390/antiox11020257

**Published:** 2022-01-28

**Authors:** Alexander Leathem, Martin Simone, Joanne M. Dennis, Paul K. Witting

**Affiliations:** 1 Charles Perkins Centre, Faculty of Medicine and Health, School of Medical Sciences, The University of Sydney, Sydney, NSW 2006, Australia; zander.leathem@gmail.com (A.L.); msim6013@uni.sydney.edu.au (M.S.); jo-dennis@optusnet.com.au (J.M.D.); 2 School of Medicine and Dentistry, Gold Coast Campus, Griffith University, Brisbane, QLD 4215, Australia

**Keywords:** Parkinson’s disease, neurodegeneration, oxidative stress, cyclic nitroxide, mitochondrial dysfunction, reactive oxygen species

## Abstract

The cyclic nitroxide TEMPOL exerts anti-oxidative and anti-inflammatory effects, and thus may provide therapeutic benefit in Parkinson’s disease (PD), in which mitochondrial dysfunction, oxidative damage and inflammation have been implicated as pathophysiological mechanisms underlying the selective loss of dopaminergic neurons. Markers of oxidative stress and inflammation were investigated in a cell model of differentiated human neuroblastoma (SH-SY5Y) cells treated with the neurotoxin, 6-hydroxydopamine (6-OHDA). Treatment with TEMPOL ameliorated 6-OHDA-mediated cytotoxicity and attenuated biomarkers of oxidative stress including: mitochondrial superoxide anion free radical production, lipid peroxidation, induction of heme oxygenase 1 (HO-1) protein expression and NFκB activation. Treatment with TEMPOL abated decreased gene expression of DRD2S and DRD2L induced by 6-OHDA indicating that TEMPOL may prevent mitochondrial dysfunction and activation of pathways that result in receptor desensitization. 6-OHDA insult decreased gene expression of the antioxidant, SOD-1, and this diminution was also mitigated by TEMPOL. Activation of NFκB increased pro-inflammatory IFNy and decreased IL-6, however, TEMPOL had no effect on these inflammation mediators. Overall, this data suggests that cyclic nitroxides may preserve dopaminergic neuronal cell viability by attenuating oxidative stress and mitochondrial dysfunction, but are unable to affect inflammatory mediators that propagate cellular damage and neurodegeneration in PD.

## 1. Introduction

Parkinson’s disease (PD) is a progressive neurodegenerative disorder that arises due to a complex interplay of age, genetic and environmental risk factors. Pathological features of PD include selective loss of dopaminergic neurons in the substantia nigra pars compacta and the presence of α-synuclein-containing Lewy bodies [1], which contribute to reduced dopamine modulation of basal ganglia functions [2] and manifest as characteristic motor symptoms of PD. Treatment of PD is primarily concentrated on the use of levodopa and deep brain stimulation, although these treatments only address symptoms [3]. Accordingly, there is a growing urgency to develop new therapeutic approaches to alter the underlying pathogenesis of the disease.

The mechanism (s) of dopaminergic neuron loss and corresponding impaired motor function is unclear. While genetic risk polymorphisms are present in familial cases, PD remains a multi-factorial and largely idiopathic disease [4]. Oxidative stress is considered a convergent point for a raft of molecular events including mitochondrial dysfunction, inflammation, genetic mutations, and aberrant protein formation; all factors implicated in PD pathogenesis [4,5]. For example, oxidized biomolecules and decreased antioxidants, such as glutathione and superoxide dismutase (SOD), are evident in the substantia nigra tissue of PD patients [6,7,8,9], with significant alteration in SOD activity also demonstrated in the PD brain [10]. Oxidative stress also disrupts the ubiquitin-proteasomal system, leading to an accumulation of damaged/misfolded proteins, another feature of PD [11].

Dopaminergic neurons are sensitive to reactive oxygen species (ROS) produced by intrinsic dopamine metabolism [12]. In addition, mitochondrial dysfunction yields intracellular oxidative stress and accordingly, is potentially a causative mechanism in PD pathogenesis. Herbicides associated with aspects of PD aetiology, such as paraquat, rotenone and 1-methyl-4-phenylpyridinium (MPP+), induce PD-like symptoms by inhibiting mitochondrial complex I, resulting in enhanced ROS production [13]. Similarly, α-synuclein, a key protein in PD pathology, can interact with mitochondrial membranes and inhibit complex I. Furthermore, mutations in genes involved in mitochondrial respiratory complex and antioxidant activity have been identified in familial PD [14,15,16]. Taken together, this data supports the underlying role of mitochondrial dysfunction in the pathogenesis of PD [17,18,19].

Increased accumulation of iron in the substantia nigra is a feature of normal aging, however, the release of soluble ferrous iron from ferritin stores is amplified in PD patients [20,21,22,23]. Unbound ferrous iron exerts its neurotoxicity by several mechanisms, including reacting with hydrogen peroxide in the Fenton reaction to generate ROS hydroxyl radicals, as well as the production of neurotoxins such as 6-hydroxydopamine (6-OHDA) [24,25]. 6-OHDA induces parkinsonian symptoms in experimental models through dopaminergic neuron damage attributed to potent inhibition of complexes I and IV, intra- or extra-cellular autooxidation and interaction with monoamine oxidase to produce ROS, which all lead to decreased neuronal cell viability [24,26].

A neuroinflammatory response is associated with PD pathophysiology [27] and damaged dopaminergic neurons in the substantia nigra release post-translationally oxidized proteins such as α-synuclein and neuromelanin, that act as inflammatory mediators [12]. Microglial activation can facilitate ongoing inflammation and neurodegeneration by producing ROS and inducing nitrosative stress [28,29]. Under such conditions, dopamine may be oxidised to mediators that inhibit mitochondrial respiration [30] and reinforce a loop of ROS production, chronic inflammation, and progressive neurodegeneration.

Contemporary PD treatments do not prevent or reverse neurodegeneration, and accordingly, there is a strong need to develop new approaches which address the underlying disease process. Cyclic nitroxides are stable free radicals with dual antioxidant and anti-inflammatory mechanisms of action [31]. Specifically, cyclic nitroxides act as a SOD mimetic to degrade ROS such as superoxide anion radicals, inhibit Fenton reactions involving free ferrous iron by reducing hydrogen peroxide bioavailability, and act as general scavengers of ROS to reduce oxidative stress and associated inflammation [31,32]. Significant research has identified numerous non-toxic and bioavailable nitroxides with antioxidant activity in various disease models [31,33,34,35,36]. Nitroxides are a promising therapeutic approach to PD with potential neuroprotective effect, as their extensive antioxidant mechanisms may inhibit cell damage and death, thereby interrupting the cycle of oxidative stress, inflammation and neuronal cell death.

In direct support of the potential therapeutic action of nitroxides, 4-hydroxy-2,2,6,6-tetramethylpiperidin-1-oxyl (TEMPOL) has been shown to reduce neurotoxicity and the severity of parkinsonian symptoms in mice following intrastriatal administration of 6-OHDA, as well as 6-OHDA-induced apoptosis in dopaminergic mesencephalic cells [37]. Similarly, TEMPOL inhibits 6-OHDA mediated decline in mice activity, as well as cytotoxicity and NFkB activation in undifferentiated PC12 cells [38]. Furthermore, TEMPOL has also been shown to reverse MPTP induced nigrostriatal dopaminergic degeneration in mice deficient in the protein apoptosis-inducing factor, a mitochondrial protein essential to the function of complex I [39]. The mechanism by which TEMPOL demonstrates a neuroprotective effect was investigated, herein in a cell model of Parkinson’s disease, by examining biomarkers of oxidative stress and inflammation induced by 6-OHDA in differentiated human neuroblastoma SH-SY5Y cells.

## 2. Materials and Methods

### 2.1. Cell Culture and Differentiation into Neuronal Phenotype

Human neuroblastoma SH-SY5Y cells (ATCC CRL-2266, passage number 5–8) were maintained in Dulbecco’s Modified Eagle’s Medium/Nutrient Mixture F-12 Ham (DMEM/F12) supplemented with 10% (*v*/*v*) foetal bovine serum (FBS), 100 U/mL penicillin/streptomycin, 2 mM L-glutamine and 1X minimum essential medium (MEM) non-essential amino acid (NEAA) solution (complete media) in a humidified incubator (37 °C, 5% CO_2_). Cells were sub-cultured upon reaching 70–80% confluency (~every six days) by addition of 0.25% (*v*/*v*) trypsin-EDTA (5 min, 37 °C). All experiments were performed with cells at a passage number <15, as these cells remained most responsive to the method of differentiation outlined below.

**Figure 1:**
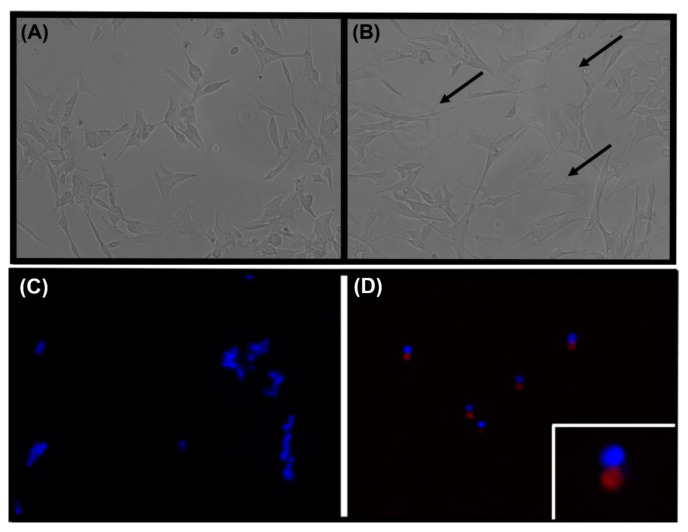
Microscopic images of native and differentiated SH-SY5Y cells under transmission Panels (**A**,**B**) and immunocytochemistry for Tyrosine Hydroxylase Panels (**C**,**D**) to confirm the anticipated dopaminergic phenotype. Cultured SH-SY5Y cells were exposed to (**A**) sterile H_2_O (vehicle control) or (**B**) RA and TPA (**B**) to initiate differentiation as described in the Methods section. After 7 days, images were captured using the EVOS FLoid Cell Imaging Station and are representative of n = 3 independent cell cultures. (**A**) Undifferentiated SH-SY5Y cells exhibited growth in multilayered clumps and lacked axonal growth. In comparison, (**B**) differentiated SH-SY5Y cells featured long dendritic processes (identified with arrows) and evenly distributed growth in a distinct monolayer typical for neuronal-like cells. The presence of tyrosine hydroxylase (red fluorescence) in native (**C**) and differentiated (**D**) cells was assessed by immunocytochemistry using a polyclonal anti-TH antibody. Cell nuclei were counterstained with DAPI (blue fluorescence). Images were captured at 20× over 5 different fields per slide and are representative of at least 5 fields of view using n = 2 independent cell culture experiments. Inset in panel (**D**) shows a computer magnified image of a TH positive cell indicating the expression of this dopaminergic phenotypic marker.

For differentiation into a neuronal phenotype, SH-SY5Y cells were seeded into 6 well plates at a density of ~225,000 cells/well in 2 mL complete media and maintained overnight in a humidified incubator. Cell differentiation was initiated with proliferating cells at ~60–70% confluence by addition of 10 μM retinoic acid (RA) in 2.5% FBS/media ( *v*/*v*) 3 days and replaced with media containing 10 μM RA and 81 nM 12-o-tetradecanoylphorbol-13-acetate (TPA) for another 3 days of subculture. Th extent of differentiation was monitored by microscopy and confirmed by phenotypic axonal projections and the formation of dendritic structures (Figure 1 below). All subsequent experiments were performed using cells differentiated by this method or control cells incubated with sterile H_2_O in place of RA/TPA cultured under identical conditions. Reagents were purchased from Sigma, Rowville, Australia unless otherwise stated and de-ionised, milliQ water (Bio-Rad, Gladesville, Australia) was used throughout.

### 2.2. Drug Treatments and Cell Harvesting

Differentiated SH-SY5Y cells were pre-treated with vehicle (sterile H_2_O) or 30 μM 4-hydroxy-2,2,6,6-tetramethylpiperidin-1-oxyl (TEMPOL) and incubated for 30 min before challenge with freshly prepared 30 μM 6-hydroxydopamine (6-OHDA) in complete media. Cells were incubated with drugs in a humidified atmosphere for 24 h before harvesting. Following incubation, the media (containing any detached cells) was aspirated into individual tubes and the remaining adherent cells were collected by treatment with 0.25% (v/v) trypsin-EDTA (5 min, 37 °C) and subsequent neutralisation of trypsin activity by the addition of complete media (1:1 (*v*/*v*)). This cell suspension was combined with aspirated media and centrifuged (5 min, 200× *g*, room temperature). The supernatant was removed, and the resultant cell pellet was resuspended in 150 μL of radioimmunoprecipitation assay (RIPA) buffer (150 mM NaCl, 1% (*v*/*v*) Triton-X, 0.5% (*w*/*v*) sodium deoxycholate, 0.1% SDS (*w*/*v*), 50 mM Tris, 1 mM EDTA, 1 mM egtazic acid (EGTA) containing Roche complete protease inhibitor (1 tablet/50 mL; Roche, Sydney, Australia) and Roche PhosSTOP (1 tablet/10 mL; Roche, Sydney, Australia)). This cell lysate was resuspended after passage through a 23-gauge needle three times with brief mechanical mixing. Samples were stored at −80 °C and multiple freeze thaw cycles were avoided. This procedure was repeated for all experiments unless otherwise stated.

### 2.3. Tyrosine Hydroxylase Immunocytochemistry

A suspension of SH-SY5Y cells were seeded in 8 well chamber slides at 20,000 cells/well and differentiated by sequential exposure to RA or TPA or sterile H_2_O (control cells). After 7 days, media was removed, and cells fixed with pre-chilled acetone at −30 °C for 10 min followed by washing with TBS-T (25 mM Tris, 137 mM NaCl, 4 mM KCl and 0.1% (*v*/*v*) Tween-20) for 3 × 3 min. Tyrosine Hydroxylase polyclonal antibody (rabbit Sigma-T8700) was diluted (1:50 *v*/*v* in (0.1% (*v*/*v*) Triton X-100, 1% (*w*/*v*) BSA in TBS-T)) and then added and incubated for 2 h in a humidified chamber in the dark. Next, wells were washed 3× with TBS-T. Secondary antibody (anti-rabbit goat IgG H&L Alexa Fluor 594, Abcam-ab150088) (1:200 *v*/*v*) was then added for 30 min. Wells were then washed 3× with TBS-T and counter-stained with spectral 4′,6-diamidino-2-phenylindole (DAPI; Perkin Elmer, Melbourne, Australia) (1:2000; *v*/*v*) for 5 min. The wells were washed 4× with TBS-T and chamber slides cover-slipped using fluorescent mounting medium (Dako, West Gosford, Australia) then imaged using a Zeiss Axio Scope.A1 microscope (Zeiss, Sydney, Australia) with excitation/emission 590/617 nm (Alexa Fluor 594) and 358/461 nm (DAPI).

### 2.4. Assessment of Cell Viability

For Trypan blue exclusion determinations, SH-SY5Y cells in 6-well plates were treated with varying concentrations (0–250 μM) of freshly prepared 6-OHDA in complete media. After 24 h, cells were collected, and the cell pellet resuspended in complete media. Aliquots were then diluted 1:1 (*v*/*v*) with 0.4% (*v*/*v*) Trypan Blue/H_2_O and samples (10 μL) loaded into Countess cell counting chamber slides and immediately analysed using a Countess II Automated Cell Counter (Thermo Fisher Scientific, Scoresby, Australia). Data was expressed as percentage of viable cells that excluded dye relative to vehicle-treated control cells in the absence of 6-OHDA.

### 2.5. Flow Cytometry

To further define cell viability, flow cytometry was performed with a commercial Apoptosis Detection Kit (Abcam, Melbourne, Australia) according to the manufacturer’s instructions. After 24 h drug treatment, cells were harvested using trypsin and subsequent neutralisation with 1:2 (*v*/*v*) calcium-free phosphate buffered saline (PBS) supplemented with 10% (*v*/*v*) FBS and 4 mM EDTA. Isolated cells and media containing detached cells were combined and centrifuged (5 min, 200× *g*) and cell pellets resuspended in 2 mL ice cold EDTA-free PBS. Cell suspensions were centrifuged (5 min, 200× *g*) and the cell pellets resuspended in 100 μL 1X Binding Buffer solution (supplied) containing 5 μL Annexin V-CF Blue conjugate and 5 μL 7-aminoactinomycin D (7-AAD). Cell samples were then incubated for 15 min in the dark before addition of 400 μL 1X Binding Buffer and analysis using a Gallios flow cytometer and associated Kaluza software (Beckman Coulter, Gladesville, Australia).

### 2.6. Protein Analysis using Bicinchoninic Acid Assay

Cell lysate samples were diluted 5× in H_2_O to a final volume of 10 μL in a 96-well plate on ice before addition of freshly prepared bicinchoninic acid (BCA) solution (containing 1:50 CuSO4:BCA (*v*/*v*), final volume 190 μL). The plate was sealed and incubated (30 min, 37 °C) before absorbance determinations at 562 nm using a TECAN M200 PRO plate reader (Tecan, Melbourne, Australia). Protein concentrations were determined relative to a standard curve generated using bovine serum albumin (BSA).

### 2.7. Enzyme-Linked Immunosorbent Assay

Quantification of IFNγ and IL-6 were conducted using commercial (elisakit.com, 2018, Scoresby, Australia) enzyme-linked immunosorbent assays (ELISA) according to the manufacturer’s instructions. Cell lysates were diluted 1:1.5 (*v*/*v*) with assay diluent 1B (supplied) and samples (100 μL) or standards (100 μL, 0–1000 pg/mL IFNγ/IL-6) were loaded into pre-coated 96-well plates (supplied), sealed, and incubated for 2 h in a humidified chamber. Plates were then washed 4× and incubated with detection antibody (100 μL, 75 ng/mL (anti-IL-6) or 25 ng/mL (anti-IFNγ), prepared in assay diluent 1B) for 1 h. Plates were then washed 4× and incubated with streptavidin-horse radish peroxidase (HRP) conjugate (100 μL, diluted 1:500 *v*/*v* in assay diluent 1B) for 45 min, then washed 5× and further incubated in 100 μL of tetramethylbenzidine (TMB) substrate for 15 min (IL-6) or 20 min (IFNγ), protected from light. Reaction was stopped by addition of supplied solution (50 μL) and absorbance immediately determined at 450 nm (with wavelength correction of 570 nm) using a TECAN M200 PRO plate reader (Tecan, Melbourne, Australia). All data were normalised relative to the corresponding total protein.

### 2.8. SDS PAGE and Western Blot

Proteins IL-6 and HO-1 and phosphorylated NF-κB-p65 (marker for transcriptional activation) were separated by sodium dodecyl sulphate polyacrylamide gel electrophoresis (SDS-PAGE) with gels assembled using TGX Stain-Free FastCast premixed acrylamide solutions (Bio-Rad, Gladesville, Australia). Samples containing 20 μg protein were diluted in 6 μL 4× Laemmli loading buffer (62.5 mM Tris-HCL, pH 6.8, 1% (*v*/*v*) lithium dodecyl sulphate (LDS), 10% (*v*/*v*) glycerol, 0.005% (*v*/*v*) bromophenol blue, 10% (*v*/*v*) 2-mercaptoethanol) and water to a total volume of 24 μL. Samples were then mixed, centrifuged briefly and heated (10 min, 95 °C) before loading alongside Pre-stained Protein Standards (Bio-Rad, Gladesville, Australia). Electrophoresis was performed in Tris-Glycine SDS running buffer (25 mM Tris, pH 8, 192 mM glycine, 0.1% (*w*/*v*) SDS) at 200 V, 3 A, 300 W for 45 min, and separated proteins were imaged in gel after 5 min of UV activation using a Chemidoc XRS+ System (Bio-Rad, Gladesville, Australia). This method of assessing total protein loading by imaging followed by densitometric quantification allowed normalization of the target protein band in the corresponding lane as assessed by Western blotting (described below).

Separated proteins were transferred onto Immobilon-FL polyvinylidene difluoride (PVDF) membranes (Merck, Bayswater, Australia) using a TransBlot Turbo System (Bio-Rad, Gladesville, Australia) at 25 V and 1 A for 30 min in transfer buffer (25 mM Tris, pH 8, 192 mM glycine, 20% (*v*/*v*) methanol). Following transfer, membranes were imaged, then immersed in blocking buffer (5 mL, 5% (*w*/*v*) BSA in TBS-T) for 1 h. The blocking buffer was removed and primary antibodies; IL-6 rabbit polyclonal (Bioss bs-0782R) 1:1000 *v*/*v*, HO-1 rabbit polyclonal (Sigma H4535) 1:500 *v*/*v* and NF-κB-p65 rabbit polyclonal (Sigma SAB4502069) 1:1000 *v*/*v*, prepared in 1% (*w*/*v*) BSA blocking buffer were added to membranes and incubated overnight at 4 °C. Membranes were then washed 3× in TBS-T. Secondary antibody (goat anti-rabbit IgG-peroxidase (Sigma-A6154) 1:5000 *v*/*v*, in TBS-T was added to membranes for 1 h. Membranes were washed 4× in TBS-T and immersed briefly in 5 mL Immobilon Forte Western HRP Substrate (Merck, Bayswater, Australia) before imaging using a ChemiDoc Imaging System (Bio-Rad, Gladesville, Australia). All densitometry analyses were performed using ImageJ software (freeware, NIH, Bethesda, MD, USA) with normalisation of the target protein achieved using total protein in the corresponding lane. Data is presented as a fold change in protein relative to control.

### 2.9. Gene Expression Analysis

RNA was extracted using an ISOLATE II RNA Mini Kit (Bioline, Eveleigh, Australia) according to the manufacturer’s instructions. In brief, cells were collected and lysed in 353.5 μL RLY Lysis Buffer (containing 1% (*v*/*v*) 2-mercarptoethanol). Lysates were processed according to the manufacturer’s protocol and finally RNA was eluted with supplied RNase-free water. Isolated RNA was purified of genomic DNA contamination using amplification grade DNase I (Sigma, Melbourne, Australia). Aliquots (1 μL) of DNase I (1 unit/μL) and 10X Reaction Buffer (200 mM Tris-HCl, pH 8.3, 20 mM MgCl_2_) were added to 8 μL isolated RNA and incubated for 15 min. Stop solution (2 μL 50mM EDTA) was then added and samples heated to 70 °C for 10 min. RNA concentration was determined using a NanoDrop 2000 spectrophotometer (Thermo Fisher Scientific, Scoresby, Australia). Prepared RNA was stored at −80 °C until required.

### 2.10. cDNA Synthesis

Complementary DNA (cDNA) was synthesized using a Tetro cDNA Synthesis Kit (Bioline, Eveleigh, Australia) in an Eppendorf Mastercycler Nexus Gradient Thermal Cycler (Eppendorf, Sydney, Australia). Reaction mixtures were prepared in RNase-free polymerase chain reaction (PCR) tubes, containing: 200 ng RNA, 0.5 μL oligo (dT)18, 0.5 μL random hexamers, 4 μL 2.5 mM dNTP mix, 1 μL RiboSafe RNase inhibitor (10 units/μL), 4 μL 5X Reaction Buffer, 1 μL Tetro Reverse Transcriptase (1 unit/200 μL) and diethylpyrocarbonate (DEPC)-treated water to a volume of 20 μL. Samples were heated to 45 °C for 30 min, 85 °C for 5 min and then chilled to 4 °C. cDNA samples were stored at 4 °C until required.

The optimum annealing temperatures of primers were determined by gradient RT-PCR. Reaction mixtures were prepared using MyTaq Mix (Bioline, Eveleigh, Australia), gene-specific forward and reverse primers (Table 1, final concentration 400 nM) and sample cDNA. Gradient RT-PCR was performed with initialisation at 94 °C for 2 min, followed by 35 cycles of denaturation (94 °C for 30 s), annealing (57.8–65.2 °C for 30 s; eight distinct temperatures/gene) and extension (72 °C for 1 min) followed by a final extension at 72 °C for 10 min and chilled to 4 °C. Aliquots of PCR products (12 μL) were loaded alongside 6 μL Hyperladder 1kb (Bioline, Eveleigh, Australia) onto a 2% (*w*/*v*) agarose gel containing 0.0075% (*v*/*v*) SYBR Safe DNA gel stain (Thermo Fisher Scientific, Scoresby, Australia). Electrophoresis was performed for 45 min at 90 V, 400 mA and PCR products imaged using a Chemidoc XRS+ System (Bio-Rad, Gladesville, Australia).

### 2.11. Quantitative Polymerase Chain Reaction

Quantitative PCR (qPCR) was performed using a SensiFast SYBR No-ROX Kit (Bioline, Eveleigh, Australia) in a Lightcycler 480 (Roche, Millers Point, Australia). Reaction mixtures contained sample cDNA, forward and reverse primers (400 nM) (refer to primer sequences listed in Table 1) and SensiFast No-ROX 2x Master Mix. qPCR was conducted with initial UDG activation at 50 °C for 2 min followed by Taq activation at 95 °C for 2 min. Samples then underwent 45 cycles of denaturation at 95 °C for 5 s, annealing at 61 °C for 10 s and extension at 72 °C for 15 s. A melt curve analysis was performed at 95 °C for 5 s, 60 °C for 1 min and acquisition at 97 °C for 30 s. Assessment of thermal melt curves for all qPCR products indicated that primers yielded a single product (Supplementary Figure S1). Samples were then cooled to 40 °C for 1 s. Standard curves were generated using serial dilutions of a mixed cDNA sample. Lightcycler Software 4.1 (Roche, Millers Point, Australia) was used to conduct melting curve and relative quantification analyses. Data was normalised to β-actin and presented as a fold change in gene expression relative to control samples.

### 2.12. MitoSOX Red Fluorescence Imaging

For cell imaging studies, cells were seeded in 8 well chamber slides at density 20,000 cells/well, differentiated and treated with 6-OHDA in the absence or presence of TEMPOL. After 24 h, drugs and media were removed and 200 μL 5μM MitoSOX Red (diluted in warm Hank’s Balanced Salt Solution (HBSS); Thermo Fisher Scientific, Scoresby, Australia) was added and the mixture incubated for 30 min in a humidified chamber. The wells were then washed with warm HBSS before addition of 200 μL 10 nM MitoTracker Green FM (in HBSS; Thermo Fisher Scientific, Scoresby, Australia) for 10 min in a humidified chamber. After washing with HBSS, chamber slides were imaged using a Zeiss Axio Scope.A1 (Zeiss, Sydney, Australia) with excitation/emission of 510 nm/580 nm for MitoSOX Red and 490 nm/516 nm for MitoTracker Green FM. For quantification, cells seeded in a 96-well plate at 8000 cells/well were differentiated then treated with 6-OHDA ± TEMPOL for 24 h, before addition of 200 μL of 5μM MitoSOX Red. Finally, fluorescence was determined at 510 nm/580 nm (excitation/emission) using a TECAN M200 PRO plate reader (Tecan, Melbourne, Australia).

### 2.13. Lipid Peroxidation (Malondialdehyde) Assay

A commercial malondialdehyde (MDA) assay was used to determine cell lipid peroxidation after drug treatments according to the manufacturer’s instructions (Abcam, Melbourne, Australia). Briefly, cells were lysed in 300 μL supplied lysis buffer and 3 μL supplied 100× butylated hydroxytoluene (BHT). Cell lysates were homogenised and centrifuged (10 min, 13,000× *g*, 4 °C). An aliquot of 10 mg/mL thiobarbituric acid (600 μL; prepared in 30% (*v*/*v*) glacial acetic acid) was then added to 200 μL supernatant and heated (60 min, 95 °C) before quenching on ice for 10 min. Aliquots (200 μL) were then added to a transparent 96-well plate and absorbance determined at 532 nm using a TECAN M200 PRO plate reader (Tecan, Melbourne, Australia). Sample MDA was quantified against a standard curve generated with authentic MDA (supplied) and normalised to total sample protein expressed as MDA per total protein.

### 2.14. Statistical Analysis

Statistical analysis was performed using GraphPad Prism 7.02 and tested for normality using the Brown–Forsythe test. Parametrically distributed data was analysed using ANOVA with Tukey’s post hoc test for multiple comparisons to account for both type-1 and type-2 errors in small data sets. Data that did not meet the homogeneity of variances assumption was analysed using the non-parametric Kruskal–Wallis H test with Dunn’s post hoc test for multiple comparisons. Data was presented as mean ± SEM with *p*-value < 0.05 considered statistically significant.

## 3. Results

### 3.1. Characterizing the Cell Model of Parkinson’s Disease

The phenotype of SH-SY5Y cells in culture typically displayed large, triangular cell somas, lacked dendritic processes, and exhibited rapid, multilayered growth (Figure 1A). In contrast, differentiation with RA and TPA resulted in a mature neuronal phenotype with characteristic extension of long dendritic processes and comparatively slower growth in distinct monolayers (Figure 1B). The development of axonal growth and synaptic connections mimics the microstructure of dopaminergic neurons in vivo [40]. Tyrosine hydroxylase (TH) protein expression was utilised to confirm differentiation of SH-SY5Y cells into a dopaminergic neuronal phenotype (Figure 1 C,D). Cells incubated in the presence of RA and TPA showed an increase in TH (red fluorescence; Figure 1D) compared to native, untreated SH-SY5Y cells (Figure 1C). TH expression appeared to be focally confined to the cytoplasm of the neuronal-like cells (Figure 1D inset), consistent with known intracellular localisation [41]. Expression of TH validated the differentiation protocol to transform SH-SY5Y cells into a dopaminergic phenotype for neuronal cell modelling of PD in vitro and hence this protocol was used for all studies outlined below.

**Figure 2:**
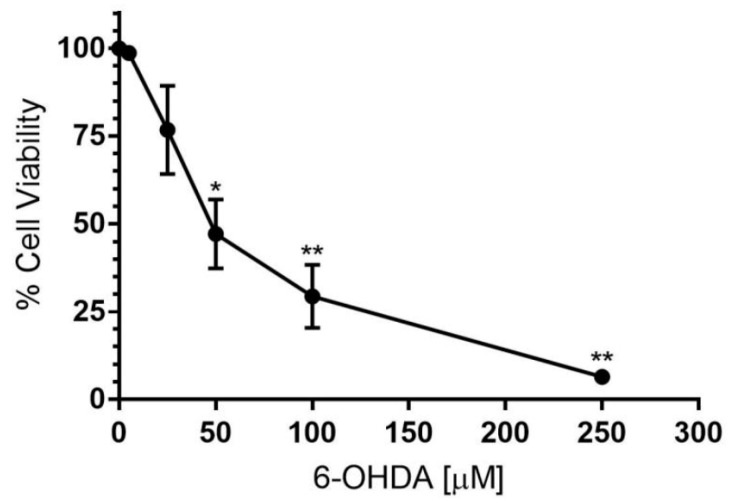
6-OHDA dose response relationship to SH-SY5Y dopaminergic cell viability. Differentiated SH-SY5Y cells were treated with increasing concentrations (0–250 μM) of the cytotoxin 6-OHDA for 24 h. Cell viability was assessed with a trypan blue exclusion assay. This viability data indicates that 6-OHDA exhibits an EC_50_ of ~50 μM in the presence of dopaminergic neuronal-like cells cultured under the reported conditions. Data represents mean ± SEM, n = 3 independent experiments. Different to the vehicle-treated control; * *p* ≤ 0.001, ** *p* ≤ 0.0001.

The neurotoxin, 6-hydroxydopamine (6-OHDA), is commonly used in experimental PD models, as it exhibits selective toxicity to dopaminergic neurons and mediates cell death by inhibition of mitochondrial respiratory complexes I and IV [24]. The dose–response relationship between 6-OHDA and differentiated SH-SY5Y neuronal cell viability was assessed initially by trypan blue exclusion. A statistically significant, dose-dependent loss in neuronal cell viability was observed for concentrations of 6-OHDA ≥ 50 μM (Figure 2). Furthermore, the data indicated an EC_50_ for 6-OHDA of ~50 μM. Therefore, a dose of 30 μM 6-OHDA, which decreased cell viability by ~30%, was selected for further experimentation without significant cytotoxicity.

### 3.2. Assessing Cell Viability on the Absence and Presence of TEMPOL

The viability of the differentiated dopaminergic SH-SY5Y cells was determined following insult with 6-OHDA in the presence or absence of TEMPOL using flow cytometry with detection of Annexin V-CF Blue/7-AAD as markers of apoptosis and necrosis, respectively. Annexin V binds to phosphatidylserine, a marker of apoptotic cells when located on the outer leaflet of the plasma membrane. In late apoptosis or necrosis, affected cells lose membrane integrity and become increasingly permeable to 7-AAD. Specific neuronal cell populations were differentiated using flow cytometry after treatment of differentiated SH-SY5Y cells with 30 μM 6-OHDA in the presence and absence of 30 μM TEMPOL, a pharmacologically achievable dose for this nitroxide [42,43].

**Figure 3:**
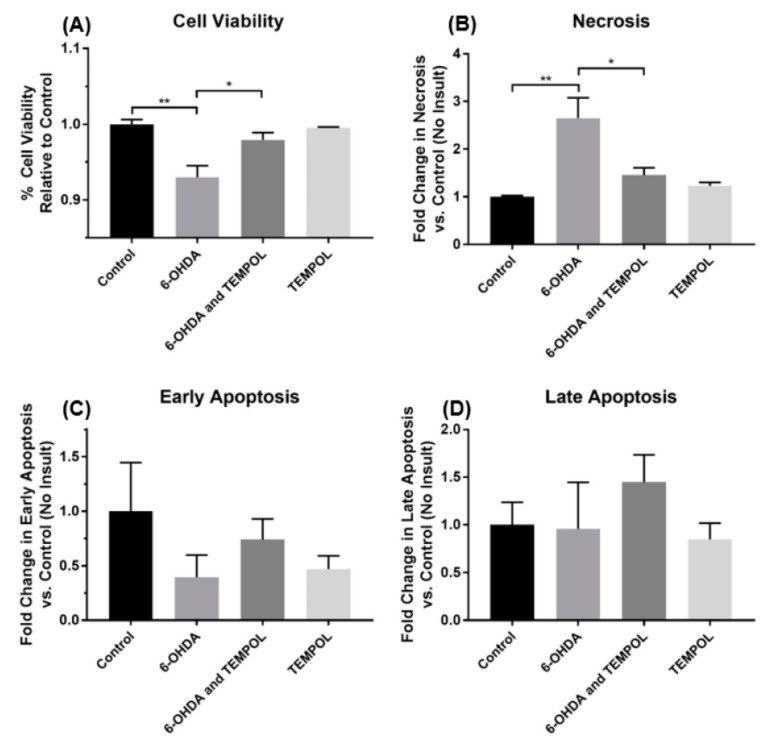
Quantification of SH-SY5Y dopaminergic cell viability after treatment with 6-OHDA ± TEMPOL. Analysis of viable cell populations was identified through analyses with flow cytometry using a commercial Annexin V-CF Blue/ 7-AAD Apoptosis Detection assay. Cultured SH-SY5Y dopaminergic cells treated with 30 μM 6-OHDA ± 30 μM TEMPOL or 30 μM TEMPOL for 24 h or untreated (control) cells were assessed for viability (**A**). Cell populations at various stages of viability; (**B**) necrotic, (**C**) early apoptopic and (**D**) late apoptopic were also quantified following the different treatments. Data represents mean ± SEM, n = 3 independent experiments. Different to the vehicle treated control or different to the cell samples treated with 6-OHDA alone; * *p* ≤ 0.05, ** *p* ≤ 0.01.

Treatment with 6-OHDA significantly reduced neuronal cell viability while pre-treatment with 30 µM TEMPOL restored viability after 6-OHDA insult (Figure 3A); TEMPOL alone did not affect SH-SY5Y neuronal cell viability. The mode of 6-OHDA mediated cell death was primarily necrosis at a concentration of 30 µM as evidenced by a 2.7-fold increase in necrosis compared to vehicle-treated control cells (Figure 3B). Treatment with TEMPOL significantly reduced necrotic cell death to ~1.5-fold that of control cells (Figure 3B). At concentrations of 30 µM, 6-OHDA did not appear to elicit apoptosis in differentiated SH-SY5Y cells (Figure 3C,D). While addition of 30 µM TEMPOL tended to increase early and late-stage apoptosis in cells treated with 6-OHDA, these changes were not statistically significant (Figure 3C,D).

**Figure 4:**
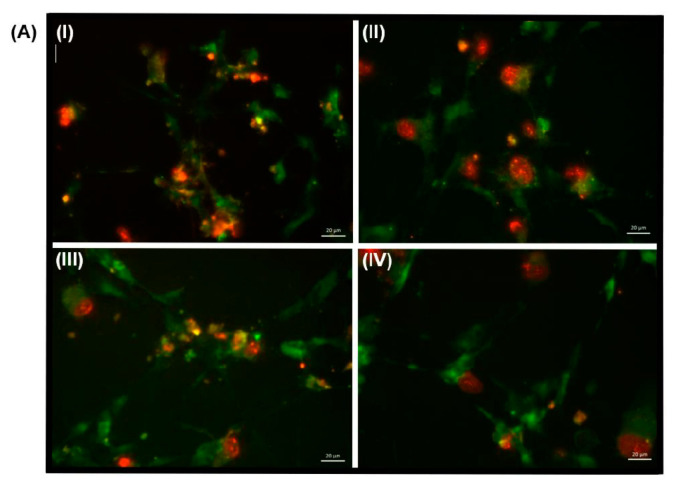

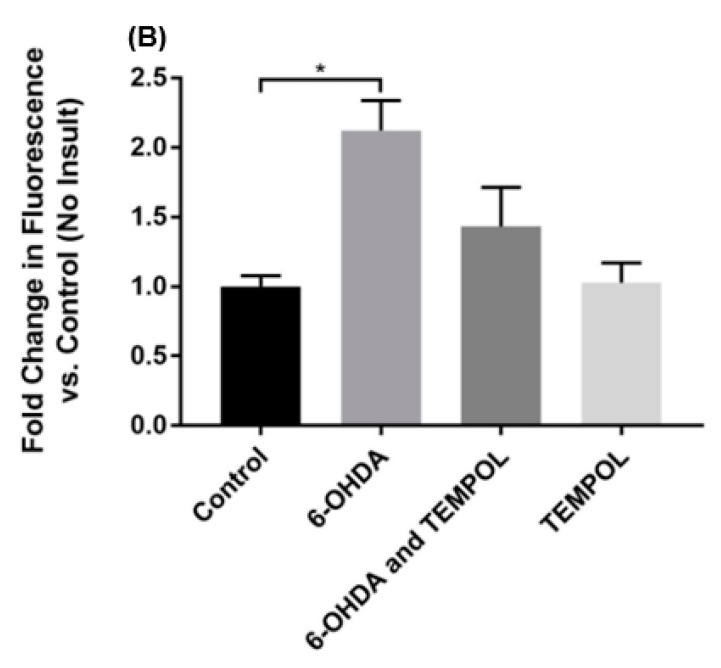
Assessment of mitochondrial superoxide anion radical production in cultured SH-SY5Y cells after treatment with 6-OHDA ± TEMPOL. Mitochondrial superoxide anion free radical production was investigated using the selective fluorescent dye, MitoSOX Red. Panel (**A**) Fluorescent microscopy panels showing cellular response after 24 h culture with (I) Vehicle (control) dopaminergic cells, (II) 30 μM 6-OHDA, (III) 30 μM 6-OHDA + 30 μM TEMPOL or (IV) 30 μM TEMPOL alone followed by subsequent incubation with MitoSOX Red and finally counterstaining with MitoTracker Green FM. Images are representative of n = 3 (each performed in triplicate) captured at 40X. (**B**) Quantification of MitoSOX Red fluorescence levels. Data represents mean ± SEM, n = 7 independent experiments. Different to the vehicle treated control; * *p* ≤ 0.05. Cells co-treated with 6-OHDA + TEMPOL or TEMPOL alone were not different to the vehicle control.

To determine whether TEMPOL improves dopaminergic SH-SY5Y cell viability following 6-OHDA insult via anti-oxidative effects, selected biomarkers of oxidative stress were assessed including superoxide anion production, lipid peroxidation and heme oxygenase-1 (HO-1) and SOD-1 expression. Mitochondrial superoxide anion free radical production was determined using the fluorescent dye, MitoSOX Red (Figure 4), which selectively targets mitochondria and exhibits red fluorescence after oxidation. Representative images demonstrated a qualitative increase in fluorescence in neuronal cells treated with 6-OHDA (Figure 4A panel II) compared to control cells (panel I). By contrast, addition of TEMPOL reduced MitoSOX fluorescence in 6-OHDA treated neuronal cells (panel III) to levels comparable to control cells. When quantified, 6-OHDA induced a significant, 2.1-fold increase in superoxide radical anion production that was inhibited by pre-treatment with TEMPOL in this cell culture model.

### 3.3. Assessment of Oxidative Damage to Cellular Lipids

**Figure 5:**
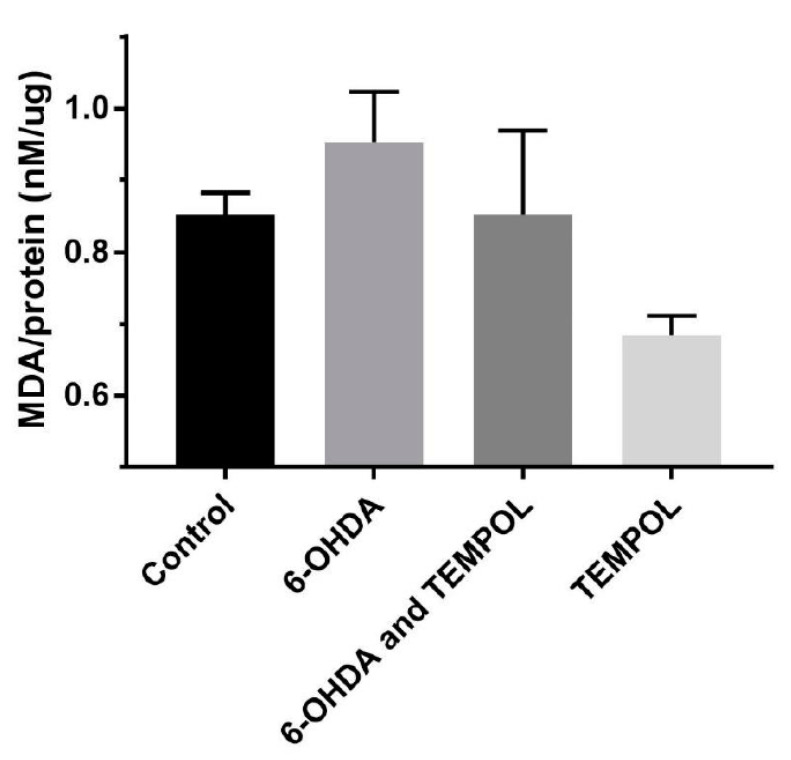
Assessment of a biomarker for lipid peroxidation in cultured SH-SY5Y dopaminergic cells after treatment with 6-OHDA ± TEMPOL. MDA levels were quantified as a biomarker for lipid peroxidation using a commercial assay kit. After 24 h of treatment with 30 μM 6-OHDA ± 30 μM TEMPOL, SH-SY5Y dopaminergic cells were harvested in assay-specific lysis buffer and MDA content determined according to the manufacturer’s instructions. Data was normalised to total sample protein and presented as mean ± SEM, n = 3 independent experiments.

Malondialdehyde is a stable end-product of polyunsaturated fatty acid lipid peroxidation and an accepted biomarker of oxidative lipid damage. Thus, MDA levels were quantified in dopaminergic SH-SY5Y neuronal cells exposed to 6-OHDA (Figure 5); MDA levels trended to increase in SH-SY5Y neuronal cells relative to control cells by ~12% (0.85 to 0.95 nM/μg protein). Treatment with TEMPOL prevented this marginal 6-OHDA-mediated increases in MDA. Furthermore, TEMPOL alone was able to inhibit MDA levels (0.68 nM/μg protein) to below that of control levels suggesting that this nitroxide may exhibit protective effects in the absence of 6-OHDA. Despite these trends, the data suggests that lipid peroxidation is not an integral pathway in the PD cell model used herein as there were no significant difference identified between treatment groups.

### 3.4. Cellular Antioxidant Response Element HO-1

**Figure 6:**
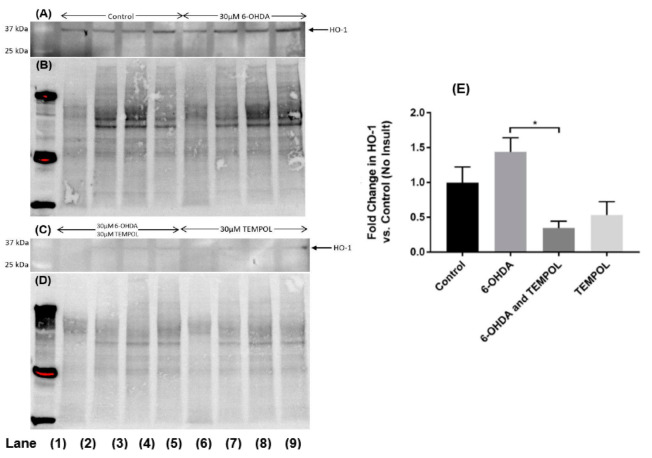
Assessment of Heme Oxygenase 1 protein expression in cultured SH-SY5Y dopaminergic cells after treatment with 6-OHDA ± TEMPOL. Cultured SH-SY5Y dopaminergic cells treated with 30 μM 6-OHDA ± 30 μM TEMPOL for 24 h were harvested and lysed before inducible HO-1 protein levels were determined by Western blotting (**A**); representative blot Lane 1: molecular weight standard; Lanes 2–5 vehicle control; Lanes 6–9; cells + 6-OHDA and (**C**) Lanes 2–5; 30 μM 6-OHDA + 30 μM TEMPOL; Lanes 6–9; cells + 30 μM TEMPOL alone). Panel (**E**) corresponding densitometric analysis of protein bands with normalization to corresponding in gel total protein assessed in the same SDS-PAGE blot (in gel protein staining shown in panels (**B**,**D**) prior to transfer step). Data is shown as mean ± SEM, n = 4 independent experiments. ^*^ Different to cell samples treated with 6-OHDA alone; *p* ≤ 0.01.

The enzyme HO-1 is induced by oxidative stress and inflammatory stimuli [44] and its protein expression in SH-SY5Y neuronal cells was assessed after 6-OHDA insult (Figure 6). Treatment with 6-OHDA alone stimulated a 45% increase in HO-1 protein expression relative to control cells. This increase in HO-1 was effectively ameliorated by co-treatment with TEMPOL, that decreased HO-1 protein to 35% of that detected in vehicle-treated control cells. Similar to the assessment of MDA, treatment with TEMPOL alone decreased HO-1 expression below the baseline level of that observed for control cells (Figure 6). This data suggests that TEMPOL ameliorates oxidative stress under both basal (vehicle-treated) conditions and cellular insult (treatment with 6-OHDA) in this experimental model of PD. The molecular weight of HO-1 is ~32 kDa however, the Western blot technique consistently identified a band corresponding to ~35–36 kDa suggestive of a post-translational modification to this antioxidant response element. Determining the nature of this modification and confirming gene regulation for HO-1 and upstream transcriptional activation (e.g., by the transcriptional factor NrF-2) warrants further investigation but was outside the scope of the present study.

### 3.5. 6-OHDA Elicits Alterations to Selected Genes

Superoxide dismutase (SOD-1) and dopamine receptor gene expression were quantified to further examine the effects of TEMPOL on oxidative stress parameters in 6-OHDA-treated neuronal cells. Altered expression and dysfunction of dopamine receptors as well as SOD-1- are pathological features of PD [45,46,47]. Gene expression changes in SOD-1, dopamine receptor D2 short isoform (DRD2S) and long isoform (DRD2L) were assessed by qPCR after 6-OHDA insult to dopaminergic SH-SY5Y cells (Table 2). Treatment with 6-OHDA significantly reduced SOD-1 gene expression by 17% relative to control cells and this reduction was significantly ameliorated by TEMPOL treatment (Table 2). Similarly, treatment of cells with 6-OHDA significantly reduced the expression of DRD2S and DRD2L by 18% and 15%, respectively, relative to control cells (Table 2). For both receptor genes, TEMPOL restored expression to levels comparable to that of control cells (Table 2). These data indicate that the altered expression of genes of key PD pathological mechanisms associated with oxidative damage is positively affected by TEMPOL.

### 3.6. Assessing Markers of Inflammation

**Figure 7:**
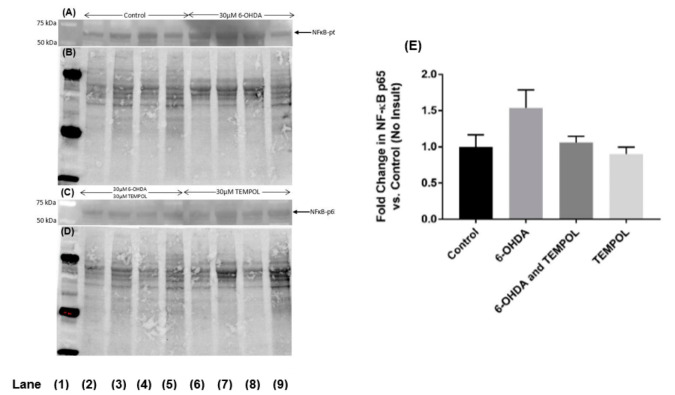
Assessment of NFκB-p65 activation in cultured SH-SY5Y dopaminergic cells after treatment with 6-OHDA ± TEMPOL. After 24 h of treatment with 30 μM 6-OHDA ± 30 μM TEMPOL, cells were harvested, lysed, and the levels of NFκB-p65 were determined by Western blotting: panel (**A**) representative blot Lane 1 molecular weight standard; Lanes 2–5 vehicle control; Lanes 6–9; cells + 6-OHDA and panel (**C**) representative blot Lane 1: molecular weight standard; Lanes 2–5; 30 μM 6-OHDA + 30 μM TEMPOL; Lane 6–9; cells + 30 μM TEMPOL alone). Panel (**E**) corresponding densitometric analysis of protein bands with normalization to corresponding in gel total protein assessed in the same SDS-PAGE blot (in gel protein staining shown in panels (**B**,**D**) prior to transfer step). Data was expressed as mean ± SEM, n = 4 independent experiments; no significant differences were determined.

Stressful stimuli including ROS induce phosphorylative activation of the transcription factor NF-κB and its subsequent translocation to the nucleus where it binds to specific gene promoters to regulate various genes involved in inflammation and cell survival. NF-κB activation in dopaminergic SH-SY5Y cells was assessed using an antibody raised against NF-κB-p65 subunit. Treatment with 6-OHDA induced a 54% increase in NFκB-p65 relative to control cells (Figure 7), albeit this did not reach statistical significance.

This increase in transcriptional activation was modulated by co-treatment with TEMPOL, which reduced NF-κB-p65 to levels comparable to the control. Treatment with TEMPOL alone further decreased NF-κB-p65 below baseline levels. While this data suggests that some degree of NF-κB activation occurs during 6-OHDA insult in neuronal cells and that this can be mitigated by the cyclic nitroxide, TEMPOL, the trends observed were not statistically significant under the conditions used here.

**Figure 8:**
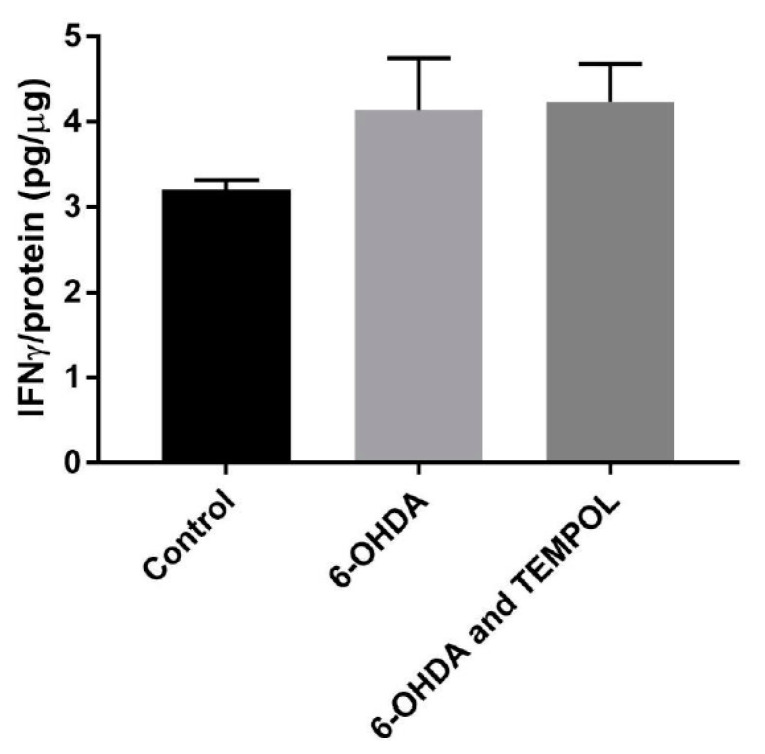
Change in IFNγ protein expression in cultured SH-SY5Y dopaminergic cells after treatment with 6-OHDA ± TEMPOL. Change in IFNγ protein expression in SH-SY5Y cells in response to treatment with 30 μM 6-OHDA ± 30 μM TEMPOL assessed with a commercial ELISA kit. Data was normalised to total sample protein and shown as mean ± SEM, n = 4 independent experiments.

Inflammation in the brain is a potential pathogenic mechanism in PD. In addition to anti-oxidative activity, cyclic nitroxides exhibit anti-inflammatory effects in cell and animal models. The effects of TEMPOL on inflammation in SH-SY5Y neuronal cells exposed to 6-OHDA was assessed. Interferon gamma (IFNγ) is a pro-inflammatory cytokine that potentiates neuronal death and activates inflammatory pathways [48] and was assessed using a commercial ELISA (Figure 8). Treatment with 6-OHDA increased IFNγ protein expression by 29% relative to control cell levels (from 3.21 to 4.13 pg/ug protein) (Figure 8). Co-treatment with TEMPOL did not alter IFNγ protein levels induced by 6-OHDA, indicating that TEMPOL did not exhibit an anti-inflammatory action against this cytokine in the cell model studied.

**Figure 9:**
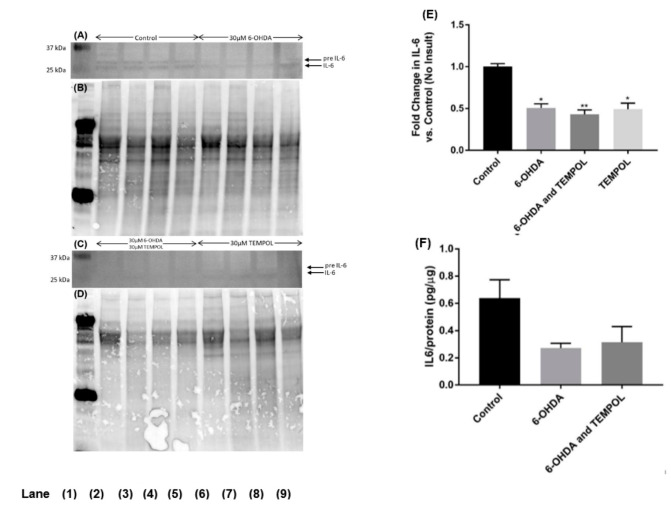
Assessment of IL-6 protein expression by Western blotting and ELISA in cultured SH-SY5Y dopaminergic cells after treatment with 6-OHDA ± TEMPOL. Levels of IL-6 protein expression were determined in SH-SY5Y dopaminergic cells 24 h after treatment with 30 μM 6-OHDA ± 30 μM TEMPOL by Western blotting: panel (**A**) representative blot Lane 1 molecular weight standard; Lanes 2–5 vehicle control; Lanes 6–9; cells + 6-OHDA and panel (**C**) representative blot Lane 1: molecular weight standard; Lanes 2–5; 30 μM 6-OHDA + 30 μM TEMPOL; Lanes 6–9; cells + 30 μM TEMPOL alone. Panel (**E**) corresponding densitometric analysis of protein bands with normalization to corresponding in gel total protein assessed in the same SDS-PAGE blot (in gel protein staining shown in panels (**B**,**D**) prior to transfer step). Different to vehicle-treated control; * *p* ≤ 0.001, ** *p* ≤ 0.0001. Panel (**F**) Levels of IL6 in cell lysates determined by commercial ELISA and data was expressed as mean ± SEM, n = 4 independent experiments; no significant differences were determined by this method.

Interleukin-6 (IL-6) is pleiotropic cytokine and demonstrates neuroprotective effects in SH-SY5Y cells [49] and was assessed in differentiated neuronal cells (Figure 9). The Western blot antibody recognised both pre-IL-6 (~28 kDa) and IL-6 (~26 kDa), hence total IL-6 protein expression was assessed by combining these two forms of the protein (Figure 9A). Treatment with 6-OHDA alone significantly reduced total IL-6 expression by ~50% relative to control cells (Figure 9A). Co-treatment with TEMPOL further reduced IL-6 expression to 43%. Notably, treatment with TEMPOL alone significantly decreased IL-6 expression to 49% of control baseline levels. A commercial ELISA was also used to assess IL-6 expression (Figure 9B). Analogous to the Western blot, treatment with 6-OHDA reduced IL-6 expression by ~50% (from 0.64 to 0.27 pg/μg protein) relative to control cells and co-treatment with TEMPOL was unable to substantially affect this decrease (0.31 pg/μg protein) (Figure 9B). This data indicates that 6-OHDA negatively regulates the expression of IL-6, and that this outcome was unaffected by the presence or absence of TEMPOL co-treatment. Interestingly, TEMPOL alone significantly diminished basal IL-6 levels confounding the interpretation of the effect of the nitroxide on 6-OHDA-treated cells studied here. Nevertheless, in combination with the IFNγ data, the combined results indicate that TEMPOL does not generally modulate inflammatory marker expression induced by 6-OHDA in cultured dopaminergic neurons, despite having positive effects on oxidative stress markers in this neuronal cell model of PD.

## 4. Discussion

The causative mechanism of selective degeneration of dopaminergic neurons in Parkinson’s disease is presently unknown. This has consequences for the design of novel therapeutic approaches; current therapies provide symptomatic relief at best and do not address the underlying pathophysiology. Neurodegeneration is thought to result from an interplay of oxidative stress, mitochondrial dysfunction, and inflammation as well as genetic mutations and abnormal handling of misfolded proteins [4]. Cyclic nitroxides have dual anti-oxidative and anti-inflammatory activities and may be a promising therapeutic approach in PD [43]. The study herein demonstrated dose-dependent necrotic cell death induced by the PD neurotoxin 6-OHDA in differentiated SH-SY5Y cells with a dopaminergic phenotype and a general neuroprotective effect in cells co-supplemented with the cyclic nitroxide, TEMPOL as judged by mitigation of cytotoxicity and oxidative stress. Thus, TEMPOL reduced mitochondrial superoxide anion production, lipid peroxidation, HO-1 protein expression and NF-κB activation. Further, TEMPOL ameliorated 6-OHDA reduced gene expression of SOD-1 and DRD2. In contrast to oxidative stress markers, TEMPOL did not exhibit anti-inflammatory effects in this cell model, as judged by an inability to affect 6-OHDA-mediated changes in IFNγ and IL-6, cytokines that are implicated in the pathogenesis of PD [48]. Overall, this data suggests that TEMPOL may provide therapeutic benefit through inhibition of oxidative stress and restoration of mitochondrial function, but is unable to affect inflammatory mediators that propagate cellular damage and neurodegeneration in PD.

The 6-OHDA dose–response relationship with respect to SH-SY5Y cell viability determined that 6-OHDA exhibited an EC_50_ value of ~50 μM. This is consistent with other similar studies, in which the reported range of EC_50_ values for 6-OHDA was ~25–100 μM [50,51,52]. Assessment of cell viability with uptake of trypan blue indicated 30 µM 6-OHDA reduced viability by ~30% however, assessment of viability by flow cytometry under the same conditions showed a decrease of only ~7%. This inconsistency is likely the result of intra- and inter-assay variability of the trypan blue exclusion assay. Notably, this small loss in viability occurred primarily via necrosis with negligible apoptosis recorded under the time and dosage conditions used here, which is comparable to another cytotoxicity study [53]. Under this relatively mild insult of 30 μM 6-OHDA, treatment with 30 μM TEMPOL inhibited the 6-OHDA-mediated decrease in cell viability to a modest, but statistically significant 2%. This neuroprotective effect is consistent with results observed in other studies, which investigated dopaminergic mesencephalic cells in vitro and cell and dopamine metabolite loss in the mouse striatum [37]. A potential mechanism for this neuroprotective effect was investigated herein.

Oxidative stress is a key pathophysiological mechanism in the neurodegeneration of PD, as dopaminergic neurons are inherently sensitive to various downstream toxic effects of ROS [12]. Here, we demonstrate that co-treatment with TEMPOL reduced 6-OHDA-stimulated mitochondrial superoxide anion production, which is consistent with its established anti-oxidative activity as a ROS scavenger and SOD mimetic [31,54]. Furthermore, elevated ROS facilitates lipid peroxidation that can induce membrane damage, secondary oxidative modification of proteins and other critical molecules and cellular injury; adverse protein modifications are associated with aging and neurodegenerative conditions [50,55]. 6-OHDA induced only a marginal increase in MDA, suggesting that lipid peroxidation is not a major pathway in the PD cell model used. However, lipid peroxidation was assessed by MDA, a product of polyunsaturated fatty acid peroxidation, and this likely under-estimated the extent of lipid peroxidation as MDA is a secondary product, is reactive and undergoes rapid enzymatic degradation [56]. Nonetheless, TEMPOL attenuated 6-OHDA-mediated increases in MDA, consistent with its reported ability to reduce lipid peroxidation by inhibiting free radical initiation and chain propagation [32,57].

Inducible HO-1 is a cytoprotective stress protein that exhibits antioxidant activity and is induced by a variety of oxidative and inflammatory stimuli [44]. Increased HO-1 expression is observed in the substantia nigra of PD and may represent a cellular response to redox imbalance and/or inflammation [58]. Pharmacological modulation of HO-1 is an active target of drug development and shows protection in models of neurodegeneration [58,59]. Although not statistically significant, HO-1 expression was substantially increased by 6-OHDA, and this change was significantly inhibited by TEMPOL. The data suggests that the 6-OHDA-induced increase in HO-1 is stimulated by ROS considering that TEMPOL exhibited anti-oxidative, but not anti-inflammatory activity in SH-SY5Y neuronal cells. Interestingly, TEMPOL, in the absence of 6-OHDA, decreased both MDA concentration and HO-1 expression relative to the vehicle-treated control, which suggests TEMPOL reduces a basal oxidant production in this cell model and thus may alleviate oxidative stress even in the absence of insult. Cyclic nitroxide reduction of oxidative stimuli may be advantageous in eliminating early mediators and pathophysiological pathways in PD.

Dysfunction of SOD, a prominent cellular antioxidant is implicated in the pathogenesis of PD and other progressive neuronal degenerative disorders [10,60]. Co-treatment with TEMPOL restored changes in SOD-1 expression induced by 6-OHDA, which indicates this nitroxide may reduce the mitochondrial dysfunction and/or oxidative stress which incites this change in SOD-1 expression. Interestingly, NF-κB activation mediates SOD expression via the PI3K protein kinase B pathway [61]. While 6-OHDA tended to increase NF-κB activation herein, its addition to SH-SY5Y cells reduced SOD-1 expression. Although unexpected, this is consistent with studies showing decreased SOD gene expression in nigrostriatal dopaminergic neurons of PD patients [47].

Alternative splicing of the DRD2 gene produces two distinct isoforms, presynaptic D2S (short isoform) and postsynaptic D2L (long isoform) [62]. D2S mediates synthesis and release of dopamine whereas D2L regulates dependent and independent signalling of G-proteins [62,63]. D2 receptor desensitisation is linked with increased intracellular Ca^2+^ [64] that can be stimulated by 6-OHDA-mediated mitochondrial dysfunction [65,66] and in turn can stimulate α-synuclein cleavage to fragments which aggregate in Lewy bodies [67]. Treatment with 6-OHDA reduced the expression of both DRD2S and DRD2L while TEMPOL restored gene expression to that of the vehicle-treated control. Considering that TEMPOL reduced mitochondrial superoxide anion this data suggests that TEMPOL may enhance DRD2 expression via modulation of mitochondrial ROS production. Nigrostriatal dopaminergic neuron D2 receptor expression is increased in PD, leading to denervation supersensitivity, a proposed adaption to decreasing dopamine concentrations with progressive neurodegeneration, which may contribute to the poor response to levodopa in late-stage disease [45,46]. Determining whether TEMPOL normalises D2 receptor expression in human PD is a potentially novel approach that may address the complications and limitations of chronic levodopa therapy in subjects suffering PD.

Reactive oxygen species are a prominent stimulant of nuclear translocation of NF-κB, a transcription factor that regulates immune responses and pro- and anti-inflammatory genes [68,69]. Increased NF-κB activation is observed in dopaminergic neurons, astrocytes, and microglia of the substantia nigra in PD as well as the midbrain of animals affected by MPTP-induced neurodegeneration [68,70]. Consistent with ROS involvement in 6-OHDA cytotoxicity, redox-sensitive NF-κB activation was elevated in dopaminergic SH-SY5Y cells and this was effectively attenuated by TEMPOL suggesting that NF-κB translocation to the nucleus to elicit downstream gene regulation was also ameliorated in the presence of TEMPOL.

For the purposes of this study, IL-6 and IFNγ were assessed to determine whether TEMPOL modulates inflammatory activity in a cell model of PD. IFNγ is a pro-inflammatory cytokine that sensitises SH-SY5Y cells to neurotoxins and mediates dopaminergic neuronal death via regulation of microglial activity [48,71,72]. While 6-OHDA trended to increase IFNγ expression, this was not significant and was unaffected by TEMPOL. IL-6 is a pleiotropic cytokine with pro- and anti-inflammatory mechanisms of action mediated by different signalling pathways [73]. IL-6 levels are positively correlated with PD severity, indicating that this cytokine acts through pro-inflammatory signalling pathways in this disease [74]. However, IL-6 has also been found to be neuroprotective in SH-SY5Y cells via activation of an acute phase anti-inflammatory signalling cascade [49]. Herein, TEMPOL had a minimal effect on the significant decrease in IL-6 levels induced by 6-OHDA in SH-SY5Y cells. This suggests that TEMPOL may not affect the chronic inflammation that propagates progressive neurodegeneration in PD [30] and any activity may be limited to its demonstrable antioxidant activity.

The differentiated neuroblastoma SH-SY5Y cell line is commonly used in PD cell culture models as it shows moderate activity of critical enzymes in dopamine synthesis and metabolism, however, there are a number of limitations associated with this cell model and the study design implemented herein [75]. Interpretation of the potential anti-inflammatory action of TEMPOL obtained here using isolated TH-positive neuronal-like cells must consider the absence of other immune cells that are present in the PD brain. Furthermore, due to its cancerous origin, there are concerns about potential genetic aberrations in the SH-SY5Y cell line, although most genes and pathways affected in the pathogenesis of PD remain intact [76]. Sequential treatment of SH-SY5Y cells with RA and TPA induces differentiation to a neuronal phenotype which more closely resembles dopaminergic neurons in vivo [76]. Importantly, in this study, differentiated SH-SY5Y cells also expressed intracellular TH, the rate-limiting enzyme in catecholamine synthesis and considered a gold standard marker for a dopaminergic neuronal phenotype [41]. However, the differentiated SH-SY5Y cell model poorly reflects the complexity of inflammatory processes of human PD that results from microglial activity [28], and this may have impacted this study’s ability to observe nitroxide effects on inflammatory markers. Further investigation with a co-culture model of microglia and dopaminergic neuronal cells, as well animal models of PD, may aid in determining the potential anti-inflammatory benefit of cyclic nitroxides [77]. Furthermore, pharmacological applications of nitroxides have limitations, for example, long term exposure and high doses of TEMPOL have been shown to impact iron transport [78]. Nonetheless, nitroxides are able to cross the blood–brain-barrier and accumulate in brain tissue, and strategies to enhance nitroxide entry to the brain are available [79,80], including incorporation into nanoparticle delivery vehicles [81].

A recent study demonstrated that the free nitroxides, TEMPO and 4-amino TEMPO, and nitroxide-containing nanoparticles, protected undifferentiated SH-SY5Y cells against 6-OHDA toxicity via attenuating ROS and mitochondrial dysfunction [33]. The data presented herein corroborates the anti-oxidative activity of this class of cyclic nitroxides. However, distinct from the Pichla et al. study [33], the data presented here employs differentiated SH-SY5Y cells with a dopaminergic phenotype confirmed by assessment of TH expression; therefore, the cell model more closely mimics neurons in the affected PD brain. A further comparison of these studies indicates that independent markers of oxidative stress were considered herein, however, in both studies nitroxides reduced levels of mitochondrial-derived oxidant production induced by 6-OHDA. Furthermore, this present investigation extended to markers of inflammation, although TEMPOL showed minimal anti-inflammatory activity. Overall, evidence provided here indicates supplemented TEMPOL may provide neuroprotection through modulation of oxidative stress rather than anti-inflammatory activity, an outcome consistent with data reported by Pichla et al. [33].

## 5. Conclusions

TEMPOL maintains cell viability via anti-oxidative and anti-inflammatory mechanisms in various disease models [31,36]. Whether TEMPOL is beneficial in PD without exhibiting a direct anti-inflammatory activity could not be ascertained from results in this study using the differentiated SH-SY5Y cell model. However, nitroxides may positively impact inflammation indirectly by reduction of ROS and ROS-stimulated pathways (including diminished NF-κB activation) and importantly, may act on other cell types in addition to neurons to yield multifaceted neuroprotection. Herein TEMPOL restored cell viability and reduced oxidative stress and associated mitochondrial dysfunction induced by 6-OHDA in a differentiated neuronal cell model of PD, consistent with a potential therapeutic benefit in PD through the amelioration of factors linked to oxidative stress. Whether other synthetic cyclic nitroxides with improved antioxidant activity/and or incorporated in novel delivery systems can show enhanced protection in vivo is yet to be rigorously demonstrated by independent laboratories. Overall, the data obtained here suggest that further development and testing of novel cyclic nitroxides may be a useful strategy to identify new potential therapies for combating PD.

## Figures and Tables

**Table 1:** List of primer sequences and NCBI reference sequences identifying primer pairs ^a^.

Gene	Forward Primer	Reverse Primer	NCBI Reference
SOD1	GGTGTGGCCGATGTGTCTAT	CACCTTTGCCCAAGTCATCT	NM_000454.4
DRD2S	GGACTCAATAACGCAGACCAGAA	CGGGCAGCCTCCTTTAGT	NM_016574.3
DRD2L	GGACTCAATAACGCAGACCAGAA	GGTGAGTACAGTTGCCCTTTAGT	NM_000795.3
β-actin	TTCCTTCCTGGGCATGGAGT	CCAGGGCAGTGATCTCCTTC	NM_001101.3

^a^ Forward and reverse primer sequences for the specified gene of interest are collected here with the NCBI reference sequence obtained from using the NCBI primer design tool (Primer BLAST NIH freeware). Corresponding levels of β-actin determined in the identical cDNA sample was employed as the house keeping gene for normalisation of target gene data throughout.

**Table 2:** Relative expression of selected genes in cultured SH-SY5Y cells treated with 6-OHDA ± TEMPOL ^a^.

Gene	Control	30 μM 6-OHDA	30 μM 6-OHDA + 30 μM TEMPOL	30 μM TEMPOL (Alone)
SOD1	1.00 ± 0.01	0.83 ± 0.06 *	0.98 ± 0.02 ^#^	1.01 ± 0.02
DRD2S	1.00 ± 0.01	0.82 ± 0.06 *	0.96 ± 0.01	0.98 ± 0.02
DRD2L	1.00 ± 0.01	0.85 ± 0.07	0.99 ± 0.02	0.99 ± 0.02

^a^ Relative changes in expression of SOD1, DRD2S and DRD2L in cultured SH-SY5Y cells in response to treatment with 30 μM 6-OHDA ± 30 μM TEMPOL assessed by qPCR. Data was normalised to β-actin expression and presented as mean ± SEM, *n* = 3 independent experiments. Different to vehicle-treated control; * *p* ≤ 0.05. ^#^Different to samples treated with 30 μM 6-OHDA; *p* ≤ 0.01.

## Data Availability

The data presented in this study are available on request from the corresponding author. The data are not publicly available due to internal issues with storing large data files on approved external data platforms.

## Supplementary Materials

The following supporting information can be downloaded at: https://www.mdpi.com/article/10.3390/antiox11020257/s1, Figure S1: Melt and Amplification Curves for Gene Expression Analysis.
