# Supplementary material for: The Cyclic Nitroxide TEMPOL Ameliorates Oxidative Stress but Not Inflammation in a Cell Model of Parkinson’s Disease
Source: Antioxidants (Basel). 2022 Jan 28;11(2):257. doi: 10.3390/antiox11020257 (PMC8868255; doi:10.3390/antiox11020257)
Supplement: Supplementary file 1 [file antioxidants-11-00257-s001.zip › antioxidants-1566642-supplementary.pdf]

antioxidants

Article Supplementary Data

The Cyclic Nitroxide TEMPOL Ameliorates Oxidative Stress but Not Inflammation in a Cell Model of Parkinson's Disease

SUPPLEMENTARY DATA

Alexander Leathem [1,2], Martin Simone [1], Joanne M. Dennis [1] and Paul K. Witting [1,*]

[1] Charles Perkins Centre, Faculty of Medicine and Health, School of Medical Sciences, The University of Sydney, Sydney, NSW 2006, Australia; zander.leathem@gmail.com (A.L.); msim6013@uni.sydney.edu.au (M.S.); jo-dennis@optusnet.com.au (J.M.D.)

[2] School of Medicine and Dentistry, Gold Coast Campus, Griffith University, Brisbane, QLD 4215, Australia

* Correspondence: paul.witting@sydney.edu.au; Tel.: +61-2-9114-0524

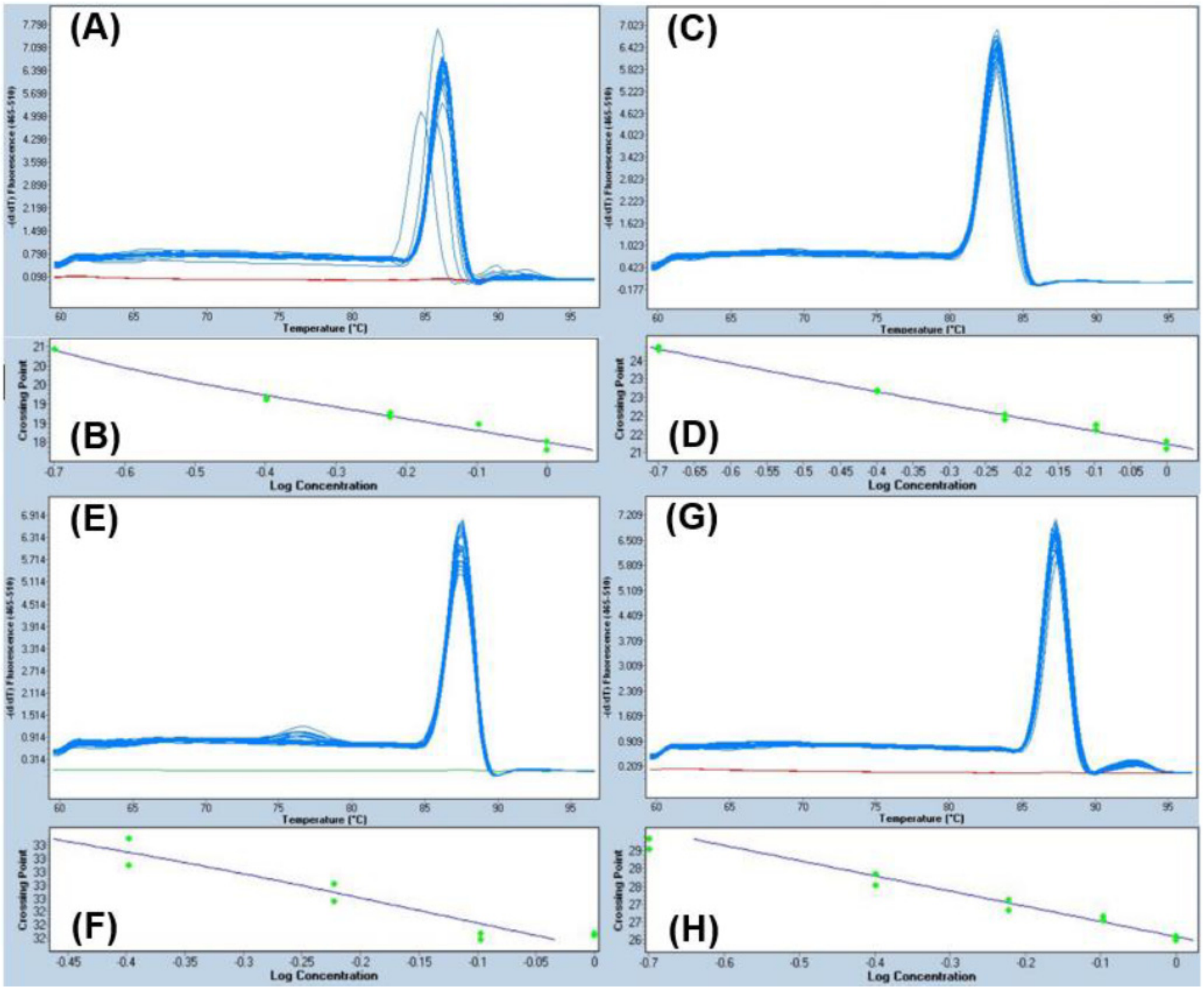

Supplemental Figure S1. Melt and Amplification Curves for Gene Expression Analysis

Serially diluted mixed cDNA samples were run alongside qPCR samples to assess amplification efficiency and primer annealing specificity. Melt and amplification curves for β-actin (A and B), SOD1 (C and D), DRD2S (E and F) and DRD2L (G and H) are shown and indicate that for the majority of samples a single product was obtained with near identical thermal melts as follows: β-actin = 86 °C; SOD1 = 83 °C; DRD2S = 87 °C and DRD2L = 87 °C.
